# Predictive analytics using Big Data for the real estate market during the COVID-19 pandemic

**DOI:** 10.1186/s40537-021-00476-0

**Published:** 2021-08-03

**Authors:** Andrius Grybauskas, Vaida Pilinkienė, Alina Stundžienė

**Affiliations:** School of Economics and Business, Kaunas University of Technology, K. Donelaičio g. 73, 44249 Kaunas, Lithuania

**Keywords:** Machine learning, TOM, Real estate, Apartments, Big data, Pandemics

## Abstract

As the COVID-19 pandemic came unexpectedly, many real estate experts claimed that the property values would fall like the 2007 crash. However, this study raises the question of what attributes of an apartment are most likely to influence a price revision during the pandemic. The findings in prior studies have lacked consensus, especially regarding the time-on-the-market variable, which exhibits an omnidirectional effect. However, with the rise of Big Data, this study used a web-scraping algorithm and collected a total of 18,992 property listings in the city of Vilnius during the first wave of the COVID-19 pandemic. Afterwards, 15 different machine learning models were applied to forecast apartment revisions, and the SHAP values for interpretability were used. The findings in this study coincide with the previous literature results, affirming that real estate is quite resilient to pandemics, as the price drops were not as dramatic as first believed. Out of the 15 different models tested, extreme gradient boosting was the most accurate, although the difference was negligible. The retrieved SHAP values conclude that the time-on-the-market variable was by far the most dominant and consistent variable for price revision forecasting. Additionally, the time-on-the-market variable exhibited an inverse U-shaped behaviour.

## Introduction

The emergence of the COVID-19 pandemic and its detrimental consequences to the global financial system were unexpected and affected millions of people by descending economic activity into a partial shutdown. Without exception, the virus reached the shores of Lithuania back on February 29th and what seemed at first to be a minuscule obstacle with a few instances of sickness reported, by March 16th the government of Lithuania folded and deliberately introduce quarantine measures shutting down almost all operations of the economy. The quarantine included restrictions and/or bans on travel, restaurants, bars, concerts, night club activities, hotels, sports clubs and tourism, leaving other leisure activities heavily regulated as well. Within these circumstances, many experts publicly claimed that housing prices would fall and assumed a 2007-style mass housing sale discount for troubled asset owners. This led to the following questions. Which prices would fall? More precisely, which predictors are best for experts to follow to anticipate price changes? Is the year when the house was built the best criterion to anticipate a discount? Or will the heating type heavily influence the price revision and should thus be monitored closely? Is it reasonable to assume that time-on-the-market (TOM) would affect the price change negatively? All these questions are extremely relevant for families, investors, entrepreneurs and even governments who are looking forward to granting financial support to harmed asset owners.

For the most part, a literature review is the beginning for data analysts in search of answers to complicated questions. Interestingly, the paradigm of the real estate theory was found to be in a predicament in many cases. For instance, the work of Johnson et al. [26], who carried out an in-depth review of previous studies addressing the price-TOM relationship, found that 29 studies had captured a positive relationship, 52 displayed a negative relationship, and 24 studies did not find any significant impact on the price. Other covariates in the literature also exhibited an omni-directional response to real estate prices, making it hard to deduce what variables influence the price revisions the most and in what direction.

Although success in various fields of using Big Data was achieved by Park and Bae [35], Borde et al. [10], Trawiński et al. [39], Čeh et al. [17], Baldominos et al. [5], De Nadai and Lepri [19], Pérez-Rave et al. [36] and Côrte-Real et al. [16], many of the mentioned papers focused on price determination hedonic models. Further, the modelling of price change in most cases was either a by-product of the models, meaning that the dependent variable was not the price change but the final transaction or listing price. Although it is easy to miss some studies in the sea of real estate literature, the ones using price change as a dependent variable were carried out by Knight [28], Khezr [27], Verbrugge et al. [40], but only probit and regression models were employed. Additionally, Pérez-Rave et al. [36] argued that the predictive power of hedonic regression is not mature and is more suited to inference, simultaneously admitting that machine learning (ML) models possess drawbacks in explaining predictive power. However, with the recent introduction of Shapley values (SHAP) created by Lundberg and Lee [31], a new dimension of knowledge can be obtained. For all of the reasons indicated in this section, this study, by using ML methods, aims to uncover the best predictors of an apartment price drop during the COVID-19 pandemic in Lithuania.

The present work makes several worthwhile contributions to the existing literature. First, it provides foresight for the households, entrepreneurs and investors who are related to the real estate sector by explaining what variables should be considered to anticipate price drops in the real estate market. Second, it provides further clarity for the TOM variable’s behaviour using the “SHAP” values. Third, it provides insight into understanding of which ML models were the most accurate for real estate predictive analytics. Fourth and finally, it contributes to the existing literature knowledge by examining feature importance in the period of pandemic.

The remainder of this paper is structured as follows. “Literature review” section analyses the existing knowledge on covariates and their implications for predicting real estate prices. “Methodology” section outlines data collection and the methodological steps taken in constructing the ML models. “Research results” section presents the empirical results and model interpretations, and “Conclusions” section provides the conclusions for the research paper.

## Literature review

Following Armstrong et al.’s [2] advice, a review of prior knowledge must be carried out before constructing a formidable forecasting model. The years of causal inference can contribute important insights and help avoid nonsensical relationships that models sometimes assign by chance, thus, to obtain a solid theoretical basis for the forecasting model, a literature review analysis was conducted in three parts. First, a review of previously used variables and their effects on price was carried out, which directed choosing candidate variables in the forecasting model. The second step examined scientific studies that attempted to measure variable importance, emphasising the literature gap. Finally, in the third step, the review of real estate and pandemic studies was discussed to gather any additional insight that could be helpful for model explanation or construction.

### The variable review

The first variable on the list was the most intriguing and widely discussed covariate among real estate scholars: the so-called TOM variable. The best summary of this variable’s effect can be described via the study completed by Benefield et al. [9], where out of 197 price equation estimations, 73 instances reported insignificant, 24—positive and 100—negative TOM relationships with the real estate price. These findings stem from two long-established theories: the search theory formed by Yinger [41] and the sale clearance theory of Lazear [29].

The former theory states that the longer a property is on the market (listed on the real estate website), the higher the probability is to discover a buyer that is willing to pay the highest price. This notion intuitively makes sense, as not all buyers are constantly refreshing websites and spotting every single property in the sea of listings. As full-time work and other personal matters consume most time for any individual, a longer TOM does not necessarily increase the likelihood of a price drop but inversely helps to find a buyer willing to pay the highest price.

In contrast, the Lazear [29] clearance model states that high TOM values for a property simply indicate a lack of buyer interest, thus, to make the property more attractive, the price needs to be reduced. The authors who sympathise with this theory argue that with longer TOM values, a certain stigma is attached to the property, as if it is not valuable or something is inherently wrong with it. The most recent papers by An et al. [3] and He et al. [25] further attempted to explain the TOM phenomenon. An et al. [3] claimed that the TOM effect on the price solely depends on the market conditions, meaning that in times of high growth, a longer TOM should help find the best buyer, but in times of economic downfall, higher TOM values will negatively affect the selling price. He et al. [25] argued that the TOM relationship is non-linear and possesses an inverted U-shaped component, meaning that up to a certain point, the TOM variable raises the chance of finding the best buyer, but after the inflection point, the TOM effect becomes negative.

Two points regarding the TOM variable must be considered. First, most of the studies tried to establish a linear model, which confines the dynamics of the TOM variable. Second, researchers have used different local market datasets. It could be that geographical locations exhibit different results. Either way, due to many differing conclusions, it is cumbersome to grasp the magnitude or the direction of the TOM variable effect while relying on earlier studies. Nonetheless, many papers consider the TOM variable an important factor influencing real estate prices; therefore, this variable is essential in the forecasting model.

The empirical findings provided by Huang and Palmquist [24], Knight [28], Anglin et al. [1], Herrin [23], Johnson et al. [26], Benefield et al. [6] and Verbrugge et al. [40] suggested that the initial price setup or the degree of overpricing can affect the price change. The idea here is that asset owners set an initial price too high with respect to other similar properties on the market and eventually have to reduce their price. This relates to information asymmetry and is acknowledged by many authors, thus, the price variable should also be included.

Another variable that is worth discussing is location. In following research papers by Rosiers et al. [37], Owusu-Edusei et al. [34], Benefield et al. [6], Khezr [27], Verbrugge et al. [40], Baldominos et al. [5], Du et al. [18], Bogin et al. [11], Metzner and Kindt [32] and Oust et al. [33], location was found to affect the price of an asset significantly in one direction or the other. The connotation behind this covariate is simply that some areas of the city have better infrastructure or perhaps higher traffic and crime rates, thus, prices are higher or lower in certain zones. Income segregation by different city zones also persists, since wealthier people tend to live in more expensive neighbourhoods. Hence, a different reaction to shocks can be expected from different areas. Some authors, such as Huang and Palmquist [24] and Park and Bae [35], even included distances to schools or shops. Families tend to look for a “full package,” meaning that the price of a building is only a part of the equation. A house might be cheaper in one zone, but if the nearest school is far away, the constant driving back and forth every month will incur additional expenses, and the initial win on a lower apartment price will evaporate in the long run. As a result, the latter variable helps to control for important factors that can affect a price change.

The huge extent of real estate literature limits the ability to review all variables; nevertheless, a pattern of many repeating covariates was detected within most studies. This included a heating type, a building type, asymmetric information, agencies, year built, proximity to shops, universities, schools, train stations, size in sq. meters, number of rooms, floors, garages, pools and other individual housing characteristics; although, little was mentioned about the significance or predictive power of each variable.

### Studies that measured variable importance

In addition to conflicting evidence as to how variables affect price, it was troublesome to extract findings on the importance or the so-called predictive power of each variable from previous studies. Surely, knowing that the TOM variable influences price changes means very little if the effect magnitude is miniscule. Unfortunately, only a handful of papers have investigated the latter issue. The papers that attempted to estimate the probability of the price change were written by Knight [28], Khezr [27] and Verbrugge et al. [40]. However, while Verbrugge et al. [40] noted that the initial rent price, TOM and location were the most important variables in predicting rent price changes, the authors regrettably did not analyse the sales price. Further, the empirical model of Khezr [27] did not provide any ranked importance but indicated that longer TOM and thin markets increased the likelihood for prices to drop. A study by Knight [28] proposed that the biggest revision was due to higher vacancy, mark-up and seller motivation. Being within a certain price range also decreased the probability for a price to change. However, the authors only employed probit or regression models, which did not address the non-linearity issues within the TOM or other variables. Moreover, recent advances in machine learning have not been tested. This leaves many answered questions and a literature gap.

### Pandemic impact on the variable importance

Regarding variable importance during pandemics, a handful of recent studies recorded that the location variable can have detrimental effects on price revisions. Liu and Su [30] discovered that during COVID-19, the housing demand shifted away from high population density areas. Similarly, Gupta et al. [22] showed that house prices and rents declined in city centres during the COVID-19 period. It is expected that people flee crowded areas, as virus infections are more likely to occur there. Even in the London cholera outbreak analysed by Ambrus et al. [4], it was reported that ten years post-outbreak, real estate prices in the city of London were still significantly lower, since a single neighbourhood had a constant reoccurring disease rate, thereby attaching a certain stigma to a particular zone. Likewise, a study published by Francke and Korevaary [20] analysed the plague outbreak in Amsterdam and the cholera spread in Paris. Both pandemics had a significant impact on population mortality rates and diminished consumer confidence, consequentially affecting the real estate market. The authors found a decline in housing prices of about 5% and around 2% in rent prices annually, it was also established that certain infected neighbourhoods lost their value due to risk perception of the renter, but these quickly reversed back after the disease disappeared. Therefore, the location or city centre variable is an important predictor.

Other studies focused more on real estate price analysis. Wong [42] recorded a small 1.5% housing price decrease during the SARS outbreak in Hong Kong. Additionally, a recent study by Giudice et al. [21] constructed a forecasting model to evaluate the COVID-19 influence on real estate price changes in Italy. The authors employed the Lotka–Volterra estimation (a “prey–predator” model) and concluded that housing prices are expected to drop by 4.16% in the short run and by 6.49% in the mid run. Following the logic of An et al. [3], the TOM variable effect should be negative since pandemics put the economy into a recession, but it could also exhibit other functional forms, as mentioned by He et al. [25].

Regrettably, the previously mentioned studies on epidemics did not yield insights into how the TOM or other variables changed and what predictive power they held during the pandemics. The location variable effect on price revision might exist, but the magnitude might be small. Also, the authors only tested regression models without trying other machine learning methods. For this reason, further empirical research is needed.

## Methodology

**Fig. 1:**
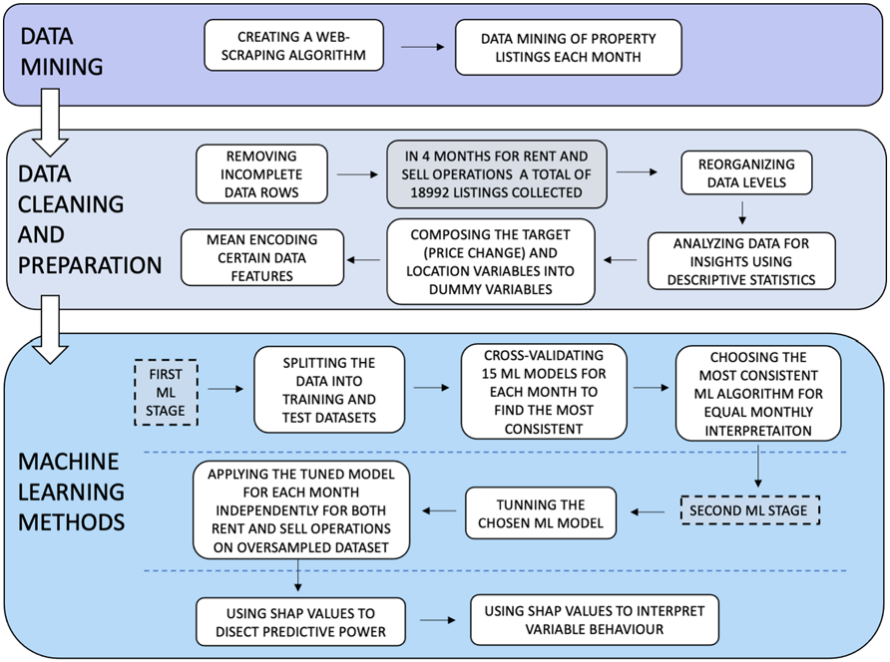
Research framework

The methodology of this paper comprised three steps: (1) data mining, (2) data cleaning and preparation and (3) machine learning methods. For better understanding, the entire research framework is depicted in Fig. 1.

### Data mining

Recently, it has become common to use a web-scraping technique for data collection. Simply put, it is a way to extract structured data from websites in an automated way and has been used by authors like Borde et al. [10], Pérez-Rave et al. [36] and Berawi et al. [7]. In this paper, the Python programming language, with packages made by BeautifulSoup and Selenium, was used to write an algorithm and purposely collect desired variables for apartment listings in the capital city of Vilnius with sell and rent operations. The data were collected monthly from May to August 2020 for a total of 4 months, and the datasets were saved independently for each month. The latter period covers two important aspects: the beginning of coronavirus, including the quarantine period, and the quarantine release period. With the quarantine restrictions increasing and decreasing, it is interesting to test whether the variables would have different impacts on the forecasting model.

### Data cleaning and processing

After the extensive data collection and cleaning procedures, a total of 18,992 apartment listings were gathered in the four-month period with at most 16 features: zone (the city zone that the apartment is located in), address, listing price, number of rooms, apartment size, the floor, the number of floors, change in the list price, year built, distance to the shop, distance to the kindergarten, distance to school, built type (whether the apartment is made of bricks, etc.), heating type, vacancy and price change date. Some features, like heating type, had more than 40 levels but were reorganised into 13 levels. It is worth mentioning that the size of the collected dataset was very close to the population size, as the retrieved data represented the majority of all existing apartment listings in Vilnius.

Afterwards, the price drops of the property listings in Vilnius were analysed and compared to previous authors’ work on pandemics. Additionally, since many authors have found the TOM variable significantly predicting price drop, a heatmap of TOM values according to the Vilnius city boroughs was created for all four months and both sell and rent operations. From the heat map, one could also observe whether vacancies were more prominent in the city centre compared to other zones, where darker colours showed higher vacancy values and brighter colours indicated smaller vacancies. Additional variable distribution visualisations of the rent and sell operations are depicted in Appendices 1 and 2.

Before applying supervised learning, data preparation and feature selection processes were initiated. First, the target variable (indicating whether a price change occurred or not) was composed into a dummy variable for each month, as follows:

1 $$I\left(y\right)= \left\{\begin{array}{ll}1, \quad y \in A\\ 0, \quad y \notin A\end{array}\right.,$$

where *I* is an indicator function with space *A* that composes dummy variable *y* into 1 if a price change occurred and into 0 if a price change did not occur. Similarly, the location variable was also composed into a dummy variable, where apartments located in the city centre were assigned a value of 1 and 0 if they were outside the city centre. Furthermore, to avoid noise and the curse of dimensionality, this study employed target encoding for the heating and built type variables. The formula for the target encoding has the following form:

2 $${\mathrm{\varphi }}^{(j)} = \frac{1}{{N}^{(j)}} \sum_{i=1}^{N}{y}_{i}+I\left\{{x}_{i}={x}^{\left(j\right)}\right\},$$

where *N* marks the collected data points (${x}_{i}$, ${y}_{i}$), *x* marks the input variables, *y* marks the target variables, *j* marks the number of levels and *I* is the indicator function that maps each level of *x* into a feature $\mathrm{\varphi }$. Additionally, particular variables like rooms, the number of floors in the building and the floor on which the apartment is located were encoded ordinally to preserve the rank order.

### Machine learning methods

The ML process had two distinct stages, as shown in Fig. 1. In the first stage, the dataset was split into 70% and 30% training and test datasets, and the most consistent ML algorithm (MCMLA) was searched on the training set between the months to ensure equal interpretation when using SHAP values, as different algorithms might exhibit different variable effects. Thus, for all four months, the following 15 algorithms were applied: CatBoost Classifier, Extreme Gradient Boosting (XGB), Light Gradient Boosting Machine, Random Forest Classifier, Extra Trees Classifier, Gradient Boosting Classifier, Linear Discriminant Analysis, Logistic Regression, Ridge Classifier, Naive Bayes, Ada Boost Classifier, K-Neighbors Classifier, Decision Tree Classifier, Quadratic Discriminant Analysis and SVM—Linear Kernel (due to an abundance of algorithms, their formulae will not be shown; however, they are standard in Python libraries). Furthermore, for each algorithm, during the stratified cross-validation, the SMOTE synthetic minority sampling algorithm was deployed on the training set, which, as described by Chawla et al. [14], considers five minority samples and calculates the nearest neighbour’s average according to the Euclidean distance metric to generate new samples. This was done for each month separately and addressed the classification bias problem.

Subsequently, the 15 models’ results for four months and both sell and rent operations were provided in seven different criteria: accuracy, area-under-the-curve (AUC), recall, precision, F1-score and Kappa and Matthews correlation coefficient (MCC). As described by Brownlee [8], in using these criteria, one can objectively choose the best models for the task at hand. In this paper, the most attention was paid to accuracy, F1 score and precision ratios since this study dealt with an imbalanced dataset with many negatives. In all cases, the higher the ratios, the better. The formula for accuracy was as follows:

3 $$\mathrm{Accuracy}=\frac{\mathrm{True\,Positives }+\mathrm{True\,Negatives}}{\mathrm{All\,Sample}},$$

which gives the general model accuracy, as it used all samples in the denominator. Meanwhile, the formula for precision in the denominator used only true positives and false positives, and had the following form:

4 $$\mathrm{Precision}= \frac{\mathrm{True\,positives}}{\mathrm{True\,Positives }+\mathrm{ False\,Positives}}.$$

As discussed by Buckland and Gey [12] and Chawla [15], there is usually a trade-off between precision and recall, as one goes up and the other goes down, thus, depending on the goal, one or the other metric can be maximised. Additionally, another measure can combine the trade-offs between precision and recall and yield a single metric of a classifier in the presence of rare cases. It is called the *F*1 metric:

5 $$F1= \frac{\mathrm{True\,positives}}{\mathrm{True\,Positives }+\mathrm{ False\,Positives}}.$$

In conclusion, the accuracy, precision and *F*1 metrics were the most important while deciding the MCMLA. Furthermore, since this paper independently analysed both sell and rent operations monthly, all models metric scores were combined and averaged. One thing to consider is that machine learning processes have a stochastic feature, meaning that in different iterations, the models changed accuracy positions [8, 38]. This is especially true when SMOTE oversampling or stratified cross-validation that splits data into different sets is used. In order to have a replicability of this paper, it was decided to set a random seed fixed.

In the second ML stage, the tuning and application of the MCMLA began. The XGB algorithm yielded the most consistent scores and was thereby chosen as the MCMLA. In the tuning process, the stratified cross-validation with the SMOTE algorithm was used again, and to achieve better precision scores, the hyperparameters of the XGB algorithm were tuned using a grid search. For the sell operations, the tuned XGB algorithm used a max depth of 8, a learning rate of 0.491 and, for the rent operations, a max depth of 8 and a learning rate of 0.41. Furthermore, to highlight the functional form of variable effects when analysing SHAP values, the SMOTE oversample method was applied to the whole dataset, and the tuned XGB model was applied independently once more each month on this oversampled dataset.

Last, the recent adaptation of SHAP values in supervised learning has opened the dimension for explainable artificial intelligence. Lundberg and Lee [31] and Christoph [13] described the principle of SHAP values as the average marginal impact of a feature value across all possible coalitions. Originally, the following formula was used in game theory to compute SHAP values:

6 $${\upphi }_{i}(v) = \sum_{S \subseteq \frac{N}{\left\{i\right\}}}\frac{\left|S\right|!\left(\left|N\right|-\left|S\right|-1\right)!}{\left|N\right|! }(v(S\bigcup \{i\})-v(S)),$$

where *v* represents a characteristic function, *S* represents a coalition, *i* represents the target variable to assess and ${\upphi }_{i}$ represents the feature contribution. In this study, the positive SHAP values pushed the prediction for price change to occur, and the negatives reduced the prediction for price changes to emerge. Furthermore, to understand the general variable predictive power, the SHAP values for each feature were averaged in absolute terms, and this number showed what predictive power on average the variable achieved among all other variables. The higher the SHAP value, the higher the predictive power. Thus, in this paper individual SHAP values and the average SHAP values will be presented.

## Research results

In accordance with previous studies on the topic of pandemics and real estate, this paper found a significant but adequate apartment price response during the COVID-19 pandemic. Within the 4-month period from May to August, only 17.2% and 10.7% of listings, on average, displayed a negative price revision in rent and sell activities, respectively, meaning that the majority of properties were intact. The price revisions for rent operations occurred after 23 days on average, while for sell operations, they occurred after approximately 63 days. Investors and brokers should pay close attention to the latter values since apartment listings over this period tend to have a higher chance of price revision. Most price adjustments aggregated in a thin left-tailed distribution with a 4-month average price drop of − 7.20% and − 4.2% for rent and sell operations, respectively (the distribution of the price change is depicted in Appendices 1 and 2). Compared to Giudice et al.’s [21] forecasting model, which predicted a 4.8% drop in the short run, and Francke and Korevaary’s [20] estimations, which recorded a 5% drop in sale prices and a 2% drop in rent prices in the case of cholera, the COVID-19 period price drop in Vilnius was similar.

When analysing the price dynamics within the four months, a pattern was observed in which the apartment price revision size tended to shrink each month, beginning in May with the largest decrease in price and ending in August with the smallest decrease in price, for both sale and rent operations. Likewise, the median prices for rent and sell operations mostly dipped in May and June, while median prices started to rise in August. Although the causal COVID-19 impact was not measured, it was recorded that the number of coronavirus cases was larger in May than in August, exactly when the biggest price dip occurred and the quarantine was still ongoing, which ended on July 16^th^. After quarantine abolition, only a few instances of viral infection were recorded; hence, businesses returned to their normal activities. The descriptive statistics for all the variables and all months are presented in Tables 1, 2, 3 and 4 for sell operations, in Tables 4, 5, 6, 7 and 8 for rent operations and also in Appendices 1 and 2.

**Table 1:** Sample descriptive statistics for month August rent operations

	N	Mean	Std	Min	Max	VIF
Number of rooms	1434	2.036960	0.917779	1.000000	6.0000	3.037466
Sq.m	1434	53.40817	29.878975	1.000000	330.00	4.514540
Apartment floor	1434	3.157601	1.921412	1.000000	9.0000	1.221069
Number of floors in the building	1434	4.905858	2.179056	1.000000	9.0000	1.295373
Year	1434	1986.223	40.109960	1092.0000	2020	1.129623
Distance to shop	1434	304.5955	348.687315	10.000000	8100	2.593864
Distance to school	1434	365.4741	363.647421	10.000000	5300	2.524234
Distance to kinder	1434	331.6806	325.059069	10.000000	5300	1.959995
Built_type	1434	0.122734	0.016241	0.083333	0.3333	1.023045
Heating	1434	0.122905	0.038970	0.000000	0.4666	1.010538
Time on the market (TOM)	1434	24.04184	25.421190	6.000000	175.0	1.039517
Initial listing price	1434	554.7672	375.138502	58.350000	3800	3.376621
If located at city center	1434	0.374477	0.484156	0.000000	1.000	1.409588
If price change occurred	1434	0.122734	0.328246	0.000000	1.0000

**Table 2:** Sample descriptive statistics for month July rent operations

	N	Mean	Std	Min	Max	VIF
Number of rooms	1474	2.035278	0.884415	1.0000	6.000000	2.819089
Sq.m	1474	53.59462	27.96496	8.0000	300.0000	4.012874
Apartment floor	1474	3.150611	1.930314	1.0000	9.000000	1.218704
Number of floors in the building	1474	4.954545	2.240156	1.0000	9.000000	1.282661
Year	1474	1988.978	28.75488	1850	2020	1.221040
Distance to shop	1474	293.2360	294.6002	10.0000	5300.0000	2.125102
Distance to school	1474	363.7516	362.1593	10.0000	4600.0000	2.067408
Distance to kinder	1474	330.9497	309.8768	20.0000	3400.0000	1.983364
Built_type	1474	0.162254	0.025613	0.1554	0.357143	1.025131
Heating	1474	0.162254	0.033542	0.0000	0.500000	1.023148
Time on the market (TOM)	1474	21.29036	27.06033	0.0000	178.0000	1.056918
Initial listing price	1474	535.6358	344.3829	95.000	3800.000	3.165490
If located at city center	1474	0.375170	0.484331	0.0000	1.000000	1.421617
If price change occurred	1474	0.162144	0.368708	0.0000	1.000000

**Table 3:** Sample descriptive statistics for month June rent operations

	N	Mean	Std	Min	Max	VIF
Number of rooms	1799	2.016676	0.88035	1.000000	6.000000	2.794912
Sq.m	1799	52.51318	27.8767	1.000000	300.000	3.501264
Apartment floor	1799	3.067815	1.7935	1.000000	9.000000	1.145140
Number of floors in the building	1799	4.801556	2.1573	1.000000	9.000000	1.198230
Year	1799	1985.669	38.7276	1521.00	2102.000	1.185635
Distance to shop	1799	298.4991	290.181	10.000000	5200.0000	1.906072
Distance to school	1799	371.4508	371.727	10.000000	4600.0000	1.944286
Distance to kinder	1799	328.0322	311.322	10.000000	4300.0000	1.645901
Heating	1799	0.186525	0.026446	0.000000	0.333333	1.022265
Time on the market (TOM)	1799	20.2779	26.6619	0.000000	176.0000	1.069220
Initial listing price	1799	518.7050	318.1839	95.000000	3000.000	2.857612
If located at city center	1799	0.375208	0.484311	0.000000	1.000000	1.395546
If price change occurred	1799	0.186215	0.389388	0.000000	1.000000

**Table 4:** Sample descriptive statistics for month May rent operations

	N	Mean	Std	Min	Max	VIF
Number of rooms	1799	1.986103	0.961625	1.000000	15.000	2.05927
Sq.m	1799	50.862696	25.88830	1.000000	196.000	2.35334
Apartment floor	1799	3.092273	1.820456	1.000000	9.000	1.11439
Number of floors in the building	1799	4.787104	2.161121	1.000000	9.000	1.14999
TOM	1799	21.105058	23.07058	1.000000	161.000	1.06832
Time on the market (TOM)	1799	519.745728	316.5270	89.0340	2500.0	2.55914
Initial listing price	1799	0.407449	0.491496	0.000000	1.0000	1.20428
If located at city center	1799	0.220122	0.414444	0.000000	1.00000	2.05927

**Table 5:** Sample descriptive statistics for month August sell operations

	N	Mean	Std	Min	Max	VIF
Number of rooms	3036	2.500000	1.076788	1.000000	20.00	3.2699
Sq.m	3036	63.12611	34.971339	11.34000	670.0	5.6493
Apartment floor	3036	3.14822	1.968558	1.000000	9.00	1.2531
Number of floors in the building	3036	4.97924	2.245383	1.000000	9.000	1.3894
Year	3036	1996.91	34.568066	1019.000	2021.0	1.3681
Distance to shop	3036	376.874	378.27094	10.00000	6000	2.3123
Distance to school	3036	455.737	429.79288	10.00000	4700	2.8402
Distance to kinder	3036	368.695	369.82084	10.00000	4900	2.2769
Built_type	3036	0.09947	0.030916	0.016129	0.222	1.0961
Furnish	3036	1.73583	0.531121	1.000000	4.000	1.2020
Heating	3036	0.09960	0.034632	0.000000	0.500	1.0803
Time on the market (TOM)	3036	45.0303	55.466930	2.000000	360.0	1.0184
Initial listing price	3036	136,281	118,123	5.900000e + 03	1,600,000	3.1374
If located at city center	3036	0.2249	0.417629	0.000000	1.000	1.3206
If price change occurred	3036	0.0994	0.299345	0.000000	1.000

**Table 6:** Sample descriptive statistics for month July sell operations

	N	Mean	Std	Min	Max	VIF
Number of rooms	3136	2.534439	1.037154	1.000000	15.0000	1.757259
Sq.m	3136	64.382672	47.938239	11.340000	1985	1.664433
Apartment floor	3136	3.164222	2.016394	1.000000	9.0000	1.285848
Number of floors in the building	3136	4.977679	2.270007	1.000000	9.000	1.427927
Year	3136	1997.441	34.164736	1061.000000	2021.000	1.423255
Distance to shop	3136	384.0082	399.657316	10.000000	6100.000	2.461288
Distance to school	3136	479.9489	462.888853	10.000000	6000.000	2.676897
Distance to kinder	3136	391.6422	426.655344	10.000000	6200.000	2.189394
Built_type	3136	0.100446	0.037032	0.029412	0.280000	1.210398
Furnish	3136	1.742666	0.609686	1.000000	4.000000	1.242468
Heating	3136	0.100510	0.047111	0.000000	0.500000	1.071377
Time on the market (TOM)	3136	43.45727	52.962261	1.000000	358.0000	1.041306
Initial listing price	3136	137,066	118,475	5.950000e + 03	1,600,000	1.967865
If located at city center	3136	0.221620	0.415403	0.000000	1.000000	1.344862
If price change occurred	3136	0.100446	0.300642	0.000000	1.000000

**Table 7:** Sample descriptive statistics for month June sell operations

	N	Mean	Std	Min	Max	VIF
Number of rooms	3335	2.517241	1.083325	1.0000	20.00	3.191328
Sq.m	3335	63.437358	35.274419	10.000	680	5.531747
Apartment floor	3335	3.184408	2.036823	1.0000	9	1.251125
Number of floors in the building	3335	4.930135	2.282712	1.0000	9.00	1.385276
Year	3335	1996	29.746126	1520	2021	1.536711
Distance to shop	3335	377	369.776639	10.000	5400	2.218792
Distance to school	3335	475.80	444.692904	10.000	6000	2.572198
Distance to kinder	3335	387.9460	398.456617	10.000	6200	1.907936
Furnish	3335	1.753523	0.608231	1.000	4.0000	1.284736
Heating	3335	0.111344	0.040510	0.0000	0.600	1.093226
Time on the market (TOM)	3335	39.294	50.558899	0.0000	354.00	1.045139
Initial listing price	3335	133,794	1.167724e + 05	5630	1,600,000	3.050077
If located at city center	3335	0.220390	0.414572	0.0000	1.00	1.379555
If price change occurred	3335	0.111244	0.314482	0.0000	1.00

**Table 8:** Sample descriptive statistics for month May sell operations

	N	Mean	Std	Min	Max	VIF
Number of rooms	2979	2.478684	1.062739	1.000000	20.000	1.821010
Sq.m	2979	56.853595	28.135194	10.000000	200.0	1.433096
Apartment floor	2979	3.148708	1.984081	1.000000	9.000	1.199743
Number of floors in the building	2979	4.896274	2.232382	1.000000	9.000	1.227095
Time on the market (TOM)	2979	31.515609	32.932334	0.000000	319.00	1.005587
Initial listing price	2979	134,638	120,524	7770	1,849,000	1.798703
If located at city center	2979	0.207452	0.405550	0.000000	1.000	1.193170
If price change occurred	2979	0.119168	0.324040	0.000000	1.0000	1.821010

**Fig. 2:**
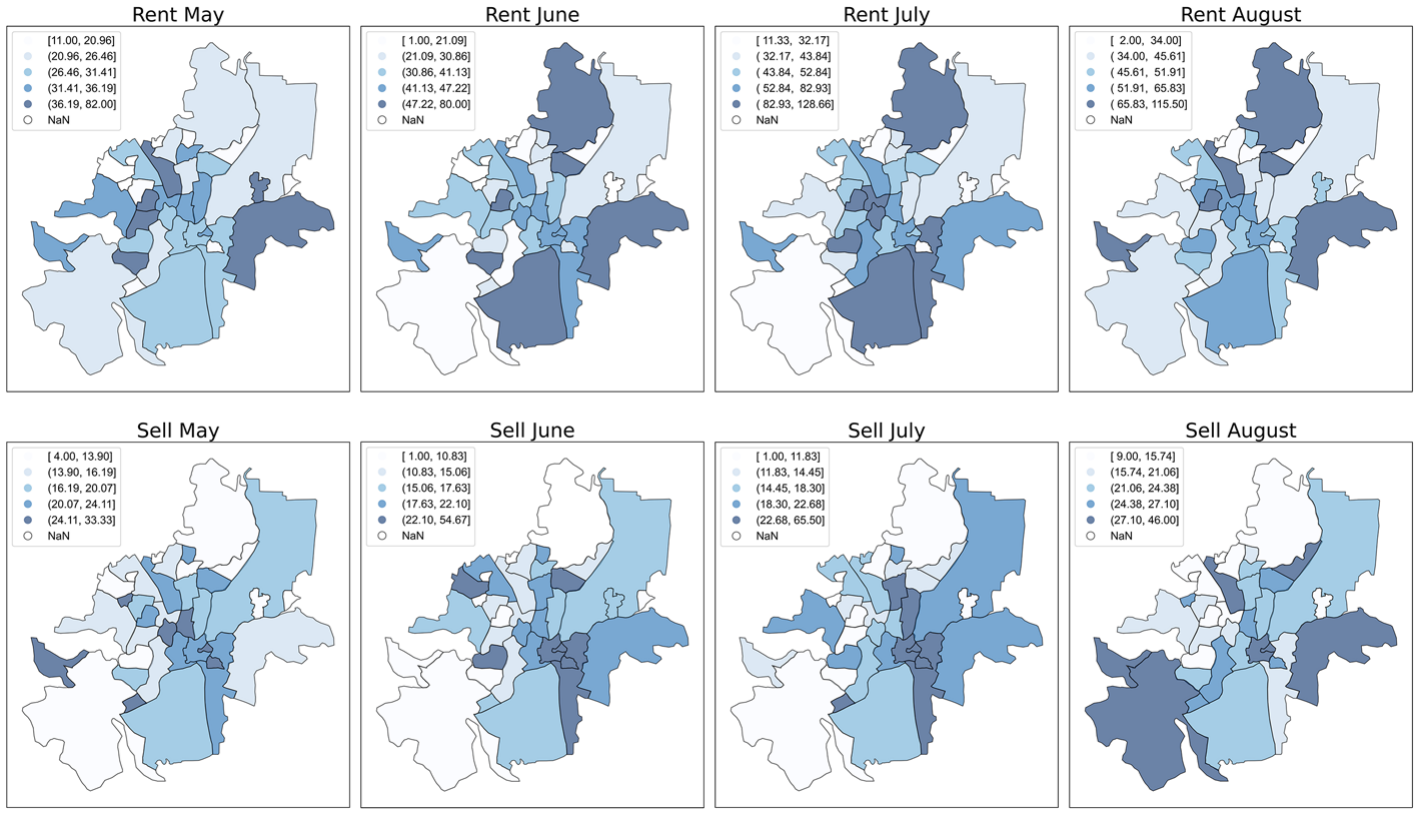
Vilnius city vacancy maps

Noticeable differences can also be observed in the vacancy rates (or the so-called TOM variable), which are depicted in Appendices 1 and 2 and Fig. 2. For rent activities, the average TOM increased from 21 to 24 days, while for sell activities, it rose from 31 to 45 days. Following the Lazear [29] clearance model, these higher TOM values would indicate that the market was in decline, as fewer buyer commitments to buy or rent were observed. With rising economic uncertainty, burdensome real estate transactions were delayed, thus, to keep their assets attractive, asset owners had to reduce asset prices or endure higher vacancies. On the other hand, the Yinger [41] theory would argue that the market participants were enduring higher TOM values to maximise their selling prices. Some believed that due to the viral spread, more crowded and denser city zones, like the old town or the new town, would endure the highest vacancies because people would start moving out to suburban areas. Unfortunately, the collected data did not validate this notion. From May to August, the vacancy growth rates for the old town and the new town increased by around 33% for sell operations, and by 11.7% and 18.8% for rent operations, respectively, although other regions underwent vacancy growth reaching up to 70% or 80%. Despite this, the city centre accounted for an average of 34.7% of rent and almost 19.1% in sell operations for all price revisions.

Finally, the 15 unique algorithms were deployed, amounting to a total of 120 machine learning models developed for each month and for both sell and rent operations. Table 9 shows the average metrics for all months and both operation types and is arranged according to the F1 column from smallest to largest. As observed, the extreme gradient boosting marginally outperformed other algorithms in F1 and accuracy metrics. For the accuracy measure, the difference between the first and second algorithms was 0.002, while for the F1 metric it was 0.022. As discussed in “Methodology” section, a trade of can be seen between precision and recall. Models that had higher precision had lower recall, and although the Catboost model had slightly better precision of 0.001, the XGB had a significantly better overall quality when looking at the F1 metric.

**Table 9:** Machine learning model results

Models		4 Months average in rent and sell operations						
		Accuracy	AUC	“Recall”	Prec	F1	Kappa	MCC
**1**	**Extreme Gradient Boosting**	**0.859**	0.782	**0.350**	**0.476**	**0.400**	**0.322**	**0.328**
2	CatBoost classifier	0.857	0.784	0.324	0.477	0.378	0.300	0.311
3	Light gradient boosting machine	0.835	0.769	0.320	0.452	0.372	0.295	0.302
4	Random forest classifier	0.851	0.779	0.323	0.453	0.370	0.289	0.297
5	Ridge classifier	0.725	0.000	0.584	0.279	0.367	0.223	0.252
6	Linear discriminant analysis	0.725	0.735	0.582	0.279	0.367	0.223	0.251
7	Gradient boosting classifier	0.834	0.767	0.351	0.385	0.364	0.268	0.270
8	Logistic regression	0.723	0.715	0.563	0.272	0.359	0.213	0.238
9	Extra trees classifier	0.851	0.780	0.305	0.455	0.358	0.278	0.287
10	Ada boost classifier	0.791	0.737	0.421	0.311	0.356	0.233	0.237
11	Decision tree classifier	0.787	0.627	0.408	0.303	0.346	0.221	0.225
12	Naive Bayes	0.520	0.666	0.692	0.198	0.293	0.101	0.139
13	K neighbors Classifier	0.671	0.614	0.478	0.209	0.288	0.116	0.133
14	Quadratic discriminant analysis	0.451	0.464	0.713	0.186	0.277	0.076	0.097
15	SVM—linear kernel	0.485	0.000	0.638	0.200	0.237	0.076	0.103

Bold values indicate the most consistent machine learning algorithm

After selecting the algorithm, eight individual models based on the XGB model were developed to dissect the feature importance by using the SHAP values (the results are depicted in Table 10). Some limitations must be noted regarding the choice of variables. Since the COVID-19 outbreak hit unexpectedly, the number of variables gathered for the first two months (May and June) was smaller compared to the number of those gathered for the last two months. Nevertheless, this study incorporated more variables with upcoming months with the intention to see if the model interpretation changed.

**Table 10:** Average feature importance of the variables according to SHAP values

Variable	Sell				Rent			
	May	June	July	August	May	June	July	August
Rooms	1.15580	0.49947	0.54986	0.55172	0.42647	0.35931	0.32831	0.46382
Sq_m_	1.60400	0.79154	0.77398	0.87373	0.44485	0.34983	0.21439	0.32318
Floor	0.52938	0.40655	0.37032	0.36983	0.17232	0.20210	0.20735	0.26256
Nr_Floors	0.65013	0.38285	0.46130	0.44349	0.18105	0.16328	0.35156	0.22225
**TOM**	**2.47043**	**2.10286**	**1.98277**	**1.88522**	**1.24815**	**1.45630**	**1.16584**	**1.12961**
Int_prices	2.28727	1.27575	1.30110	1.41305	1.26008	0.71070	0.62880	0.47666
Center	0.37411	0.30217	0.31365	0.37559	0.37768	0.41106	0.29141	0.31965
Year	–	1.54729	1.55409	1.48564	–	0.30344	0.29409	0.49686
Shop	–	0.76183	0.74752	0.94674	–	0.32995	0.38609	0.38636
School	–	0.74277	0.65204	0.65167	–	0.25219	0.31284	0.29746
Kinder	–	0.87993	0.75086	0.74460	–	0.28952	0.33553	0.37077
Furnish	–	0.20347	0.15533	0.16374	–	–	–	–
Heating	–	0.72057	0.66822	0.47049	–	0.40635	0.28597	0.47998
Built_type	–	–	1.13302	1.00620	–	–	0.57337	0.47470

Bold values indicate the most dominant variable in predicting price change

When scrutinizing the feature importance scores, a clear dominant factor was observed in both sell and rent operations over the entire four-month (May to August) period. According to the SHAP scores, the TOM variable was the single most important feature in explaining whether any price change would occur or not. The TOM variable had an average 4-month SHAP value of 2.11 for sell operations and 1.24 rent operations. While adding more variables to the models changed the TOM SHAP score, it still remained consistently the largest influencer for price revision to materialise. For the sell operations, the year and initial price setup served as the second and third largest contributors in the model, whereas other variables were far less useful at dissecting the change, especially when more variables were added; in the rent case, the predictive models relied heavily on the TOM and initial price variables, with the minimum effect from the remaining covariates.

**Fig. 3:**
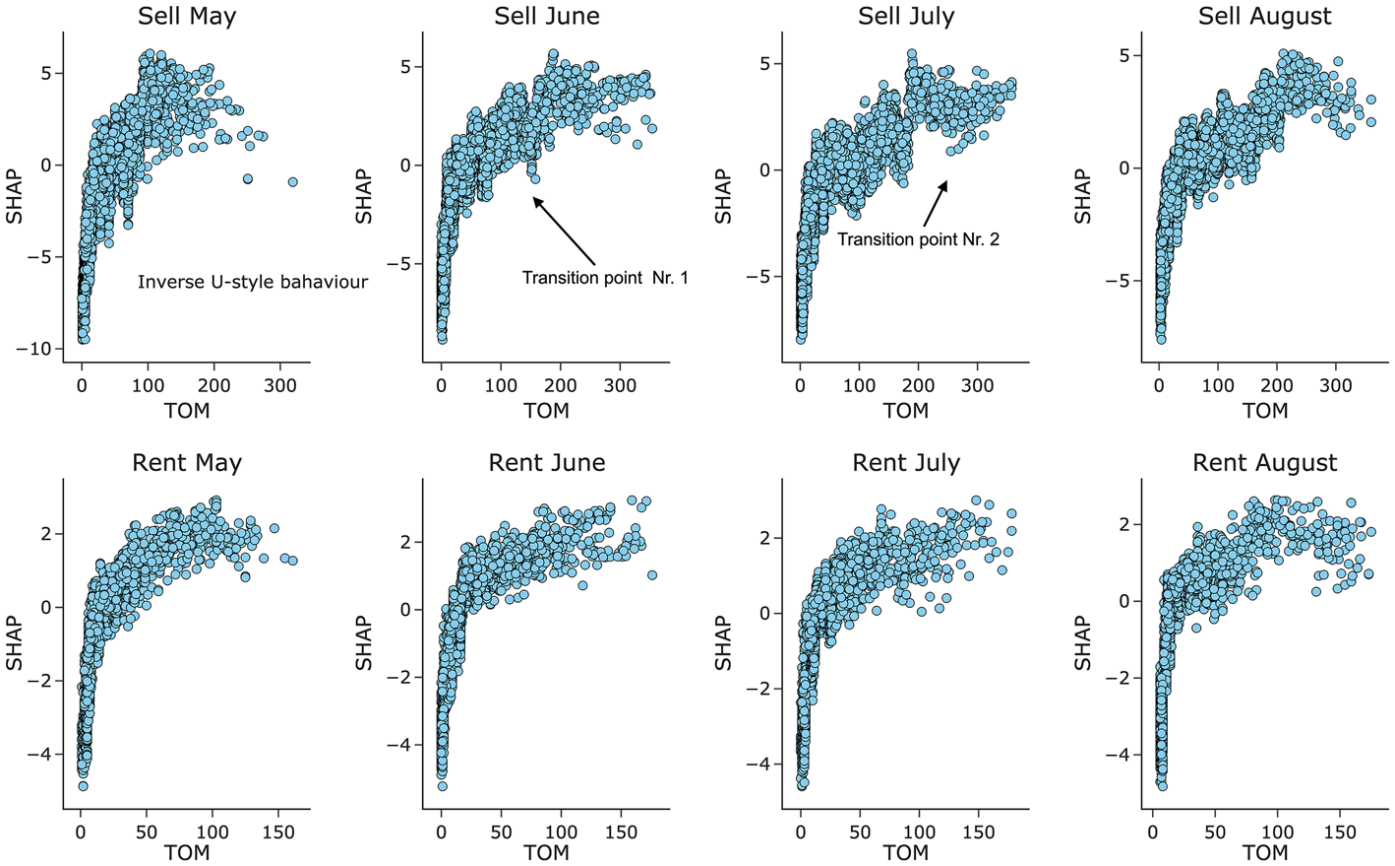
The TOM variable effect

Furthermore, it was relevant to take a closer look at the TOM variable since it demonstrated powerful capabilities for predicting future changes. The results are depicted in Fig. 3 which show individual SHAP values. Similar to He et al.’s [25] discoveries, Fig. 3’s explanation incorporates both Lazear’s [29] and Yinger’s [41] theories. As apartments were listed for a certain time duration, the TOM variable had a negative effect on the price change variable, meaning that it was not rational to expect a price change at the beginning of the listing. That is why in Fig. 3 low TOM values have negative SHAP values. Interestingly, two smooth transition points occurred later on. For the rent operations, the first smooth transition occurred after around 25 days and after around 45 days for the sell operations. From this point on, the TOM variable began to push the price revisions to occur (SHAP values became positive), but as the number of days increased, a U-style behaviour emerged, eventually leading to a second transition point where diminishing effect for price revision to occur from TOM variable was recorded. The second transition point was between 90–120 and 200–250 days for rent and sell operations, respectively. It could be that asset owners have a pre-determined limit to how much of loss they are able to bear. These findings coincide with the Lazear clearance model, which proposed that with an increase in TOM, properties begin to lose their attractiveness, and eventually, a price revision occurs, nevertheless, they also incorporated Yinger’s theory, stating that with longer waiting times, a higher chance of buyers ready to pay the highest price might occur. Additionally, the findings confirm He et al.’s [25] notion that the relationship between the price and TOM is not linear but more of an inverted U-shape, although the right-hand side of the TOM variable in Fig. 3 is less defined. Thus, entrepreneurs should base their investment strategies not on the highest TOM values, but on the range between the two transition points where the inflection occurs.

## Conclusions

The COVID-19 pandemic has dramatically affected many economic operations, and within these circumstances, real estate experts have claimed that real estate prices might fall. However, this study raised the question of what apartment attributes or variables are most likely to influence price revisions during the pandemic. In analysing the previous literature, particular variable effects were unclear on many occasions, especially regarding the TOM variable, which varied from extremely significant to not significant at all. Furthermore, many scholars focused on hedonic price determination models, while the pandemic mostly employed price change analysis. Thus, a niche for new research was discovered.

With the rise of Big Data, this study was able to create a custom web-scraping algorithm and collect property listings in the city of Vilnius during the first wave of COVID-19. Subsequently, 15 different ML models were applied to forecast apartment revisions, and each model was evaluated per particular criteria to identify the most accurate algorithm. Furthermore, the recent development of SHAP values allowed this study to dissect the variable predictive power.

The findings in this study coincide with the previous findings that real estate is quite resilient to pandemics, as the price drops were not as dramatic as anticipated. A four-month average price drop only reached − 7.20% and − 4.2% for rent and sell operations, respectively. However, an increase in apartment vacancies in most Vilnius boroughs was recorded, suggesting a worsening situation for the real estate market. Out of 15 different models tested, the XGB was the most precise, although the difference was negligible about 0.002 in accuracy criteria and 0.022 in the F1 metric. The retrieved SHAP values concluded that the TOM variable was by far the most dominant and consistent variable for price revision forecasting. Second, in line was the initial price setup. Additionally, the TOM variable exhibited an inverse U-shaped behaviour that was previously discovered by other authors, implying that there are two transition points, one at around 25 and 45 days and the other between 90–120 and 200–250 days for rent and sell operations, respectively.

From a social impact perspective, this study gives guidance to investors, households and other market participants how to evaluate the real estate market conditions and how to anticipate price revisions. For one, growing TOM values in the boroughs could indicate either emerging problems in the market that can lead to recessions or over supply of properties. Thus, governments should closely monitor TOM values as it consistently provides useful information in real time rather than waiting for monthly housing price indexes to appear. Secondly, although many variables have been found to significantly affect price change in prior studies, their effect in this study was found to be miniscule or inconsistent except for the TOM variable. Therefore, households or investors should carefully consider the TOM values when making future investments, as lower TOM values might indicate higher property resilience to market disruptions.

## Figures and Tables

**Fig. 4:**
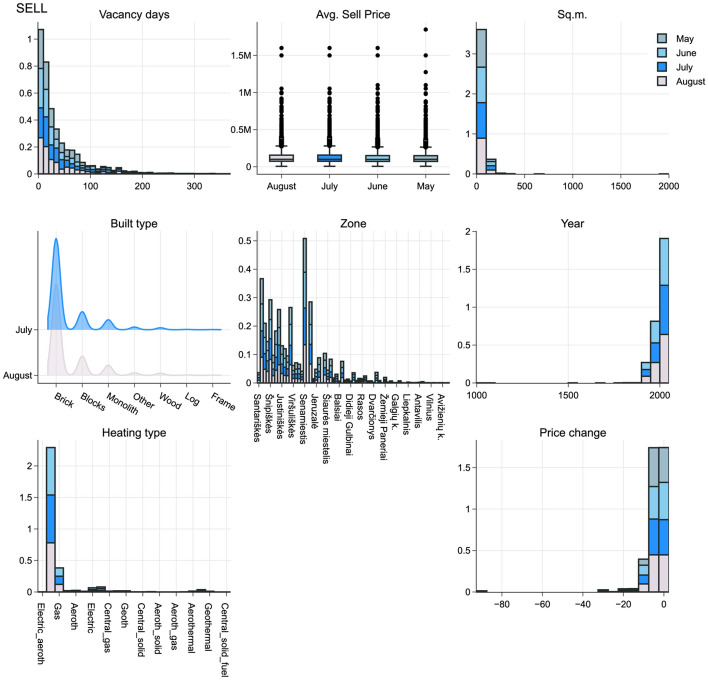
Summary statistics of sell variables

**Fig. 5:**
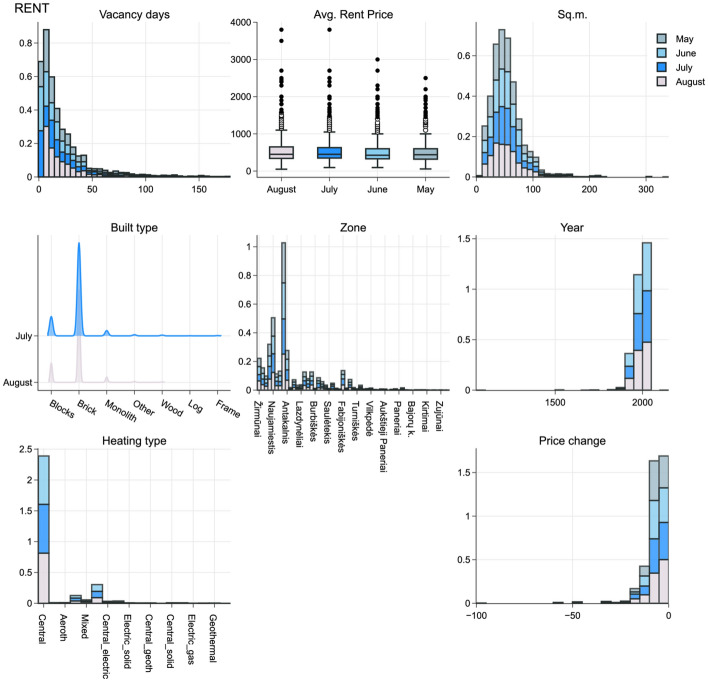
Summary statistics of rent variables

## Data Availability

The datasets generated and analysed during the current study are available from the corresponding author on reasonable request.

## Appendix 1

See Fig. 4.

## Appendix 2

See Fig. 5.

## Abbreviations

TOM: Days an apartment is listed on the market (time on the market)
XGB: Extreme gradient boosting
ML: Machine learning
MCMLA: Most consistent machine learning algorithm
